# Over-Expression of DSCR1 Protects against Post-Ischemic Neuronal Injury

**DOI:** 10.1371/journal.pone.0047841

**Published:** 2012-10-29

**Authors:** Vanessa H. Brait, Katherine R. Martin, Alicia Corlett, Brad R. S. Broughton, Hyun Ah Kim, John Thundyil, Grant R. Drummond, Thiruma V. Arumugam, Melanie A. Pritchard, Christopher G. Sobey

**Affiliations:** 1 Vascular Biology and Immunopharmacology Group, Department of Pharmacology, Monash University, Clayton, Victoria, Australia; 2 Department of Biochemistry and Molecular Biology, Monash University, Clayton, Victoria, Australia; 3 School of Biomedical Sciences, University of Queensland, Brisbane, Queensland, Australia; Rutgers University, United States of America

## Abstract

**Background and Purpose:**

The Down syndrome candidate region 1 (*DSCR1*) gene is located on human chromosome 21 and its protein is over-expressed in brains of Down syndrome individuals. DSCR1 can modulate the activity of calcineurin, a phosphatase abundant in the brain, but its influence on stroke outcome is not clear. We compared stroke outcome in wildtype (WT) and transgenic (DSCR1-TG) mice which over-express isoform 1 of human DSCR1.

**Methods:**

Transient cerebral ischemia was produced by occlusion of the middle cerebral artery for 0.5 h. After 23.5 h reperfusion, we assessed neurological impairment, brain infarct and edema volume, leukocyte infiltration and markers of inflammation. Intrinsic resistance to apoptosis following glucose deprivation was also assessed in primary cultures of WT and DSCR1-TG neurons.

**Results:**

In contrast to WT, DSCR1-TG mice had an improved neurological deficit score, greater grip strength, attenuated infarct volume and brain swelling, and lacked hippocampal lesions after stroke. Expression of mouse DSCR1-1, but not DSCR1-4, mRNA and protein was increased by ischemia in both WT and DSCR1-TG. Brain calcineurin activity was increased to a similar degree after ischemia in each genotype. DSCR1-TG mice had fewer infiltrating neutrophils and activated microglia compared with WT, in association with an attenuated upregulation of several pro-inflammatory genes. Neurons from DSCR1-TG mice were more resistant than WT neurons to apoptotic cell death following 24 h of glucose deprivation.

**Conclusions:**

Over-expression of DSCR1 in mice improves outcome following stroke. Mechanisms underlying this protection may involve calcineurin-independent, anti-inflammatory and anti-apoptotic effects mediated by DSCR1 in neurons.

## Introduction

Stroke is the second cause of death worldwide, accounting for ∼10% of all deaths, and is a major cause of long-term disability [1]. Around 80% of all strokes are classified as ischemic [2], and are due to the obstruction of blood flow to part of the brain which is thereby is deprived of both oxygen and glucose [3]. This results in the formation of an infarct consisting of an ischemic core surrounded by a penumbra. After an ischemic event the brain tissue in the penumbra undergoes a series of complex neurochemical changes termed the “ischemic cascade”, which is characterised by a depletion of energy within the cells followed by the disruption of ion homeostasis, release of glutamate, calcium channel dysfunction, oxidative stress and the induction of inflammatory responses. This complex series of events can lead to cell death through both necrosis and apoptosis [3]. While early restoration of blood flow will minimize the ultimate degree of damage, reperfusion is also associated with injury associated with an influx of various inflammatory and immune cells [4].

*DSCR1* (*aka RCAN1* [5]) is a gene located on human chromosome 21 [6]. DSCR1 mRNA and protein are elevated 2–3 fold in the brains of Down syndrome individuals compared with controls [7]. DSCR1 protein commonly exists as two isoforms, DSCR1-1 and DSCR1-4, which have identical C-terminal domains but differ at the N-terminus due to the actions of alternative promoters, leading to the inclusion of either exon 1 or exon 4, respectively [6]. Both isoforms are expressed throughout the body, however DSCR1-1 is expressed at higher levels in the brain than DSCR1-4 [8], [9]. The influence of DSCR1 on stroke outcome is not yet clear. DSCR1 is known to facilitate transient adaptation to oxidative and calcium stress and therefore could provide protection following ischemic stroke [10], [11]. For example, expression of a hamster ortholog of *DSCR1, Adapt78*, is induced by oxidative and calcium stress, and cells over-expressing this gene display greater resistance to injury [11], [12]. Following ischemia-reperfusion, brain expression of DSCR1-4 mRNA and protein is reported to increase in control animals, and infarct volume was recently reported to be modestly increased in DSCR1-deficient mice [13], [14]. DSCR1 modulates the activity of calcineurin, a Ca^2+^-calmodulin-dependent protein phosphatase abundant in the brain [15]. One of the best characterised targets of calcineurin is the transcription factor, nuclear factor of activated T cells (NFAT). Once activated, calcineurin dephosphorylates NFAT, allowing it to translocate into the nucleus where it activates the transcription of pro-inflammatory and pro-apoptotic genes [16]. DSCR1 may also exert anti-inflammatory effects by attenuating nuclear factor κ B-mediated (NFκB) transcriptional activation via stabilization of inhibitory κ Bα through a calcineurin-independent pathway [17]. Furthermore, over-expression of DSCR1 in neuroblastoma cells results in reduced expression of inflammatory markers [13].

Pharmacological modulators of calcineurin, such as cyclosporin A and FK506, reduce neuronal death following cerebral ischemia [18]. To test if increased levels of DSCR1 expression affect the outcome of ischemic stroke *in vivo*, we performed cerebral ischemia-reperfusion studies in mice that over-express the human ortholog of *DSCR1-1* (DSCR1-TG) versus wild-type (WT) control mice [19]. In addition, we compared the susceptibility of cell death in primary neuronal cultures from DSCR1-TG and WT mice following exposure to 24 h of glucose deprivation. Our findings indicate that DSCR1 is neuroprotective following ischemic stroke through mechanisms that include reduced cerebral inflammation and apoptosis.

## Materials and Methods

### Ethics statement

This study was conducted in strict accordance with the National Health and Medical Research Council of Australia guidelines for the care and use of animals in research and was approved by Monash University Animal Care and Use Committee. All surgery was performed under ketamine (80 mg/kg, i.p.) and xylazine (10 mg/kg, i.p.) anaesthesia.

### Animals

A total of 100 male 8–14 week-old mice were studied in adherence with the ARRIVE guidelines, consisting of: 51 DSCR1 transgenic (TG) mice (C57Bl/6/CBA) (weight, 23.3±0.3 g), and 49 age-matched wild-type (WT) mice (C57Bl6J/CBA) (24.8±0.6 g). DSCR1-TG mice were generated on a mixed genetic background of C57Bl6/J x CBA using human DSCR1 cDNA encoding the exon 1 splice variant (isoform 1) as previously described [19]. Mice had free access to water and food pellets before and after surgery. Twenty-five of these mice (11 WT and 14 TG) were excluded from the study because during the surgical procedure to induce stroke: a) the filament did not stay in place for the entire 30 min (n = 2); b) the occluding clamp was in place for ≥5 min (n = 2); c) the regional cerebral blood flow (rCBF) dropped to <10% of the pre-ischemic level (n = 8); d) they died prior to the specified time for euthanasia (n = 7); or e) technical or anesthetic complications arose during surgery (n = 6). Mice were randomly assigned to study groups (e.g. sham or stroke) by an investigator not performing surgical procedures or data analysis. Moreover, the investigator performing the surgical procedure or data analysis (e.g. neurological assessment, infarct volume quantification and immunohistochemistry) was usually (and wherever possible) masked to the study group to which the animal or tissue belonged.

### Blood pressure measurement

Systolic blood pressure (BP) was measured in some mice prior to cerebral ischemia via tail cuffing, using a MC4000 Blood Pressure Analysis System (Hatteras Instruments, USA). Mice were placed into individual dark chambers on a preheated platform, their tails inserted into a cuff and secured with tape to undergo 15 preliminary cycles on the day before experimental measurements were taken. On the experimental day, after five preliminary cycles, 30 measurement cycles were completed and the average value was recorded.

### Induction of cerebral ischemia

Focal cerebral ischemia was induced as described [20]. Briefly, transient (0.5 h) intraluminal filament occlusion of the right middle cerebral artery (MCA) was performed on mice anesthetized with ketamine-xylazine. rCBF in the area of cerebral cortex supplied by the MCA was monitored and recorded using transcranial laser-Doppler flowmetry (PF5010 LDPM Unit, Perimed, Sweden), beginning at the start of surgery and continuing for 0.5 h after commencing reperfusion. A midline incision was made in the neck to expose the carotid artery, a 6-0 nylon monofilament with a silicone-coated tip (Doccol, USA) was inserted and advanced distally along the internal carotid artery to occlude the MCA at its junction with the circle of Willis. Severe (∼75%) reduction in rCBF confirmed accurate placement of the filament. After 0.5 h the monofilament was retracted to allow reperfusion for 23.5 h. The laser-Doppler probe holder was kept in place following surgery, and the head wound was closed around it. Just prior to 24 h, mice were re-anesthetized, the probe was inserted and the average rCBF measured over 1 min was recorded.

### Evaluation of neurological function

Neurological assessment was performed using a five-point scoring system, as described [20], [21]. Mice were assigned a score from 0 to 4 according to the following criteria: 0, normal motor function; 1, flexion of torso and contralateral forelimb exclusively to the contralateral side when mouse is lifted by the tail; 2, circling to contralateral side when mouse is held by the tail on a flat surface, but normal posture at rest; 3, leaning to contralateral side at rest; 4, no spontaneous motor activity or rolling. A hanging wire test was also performed to test motor function, gripping ability and forelimb strength [22]. Mice were placed so that their front paws were gripping a wire 30 cm above soft padding. The time suspended from the wire, up to a maximum of 60 s, was measured and the average of 3 trials with 5 min rests in between recorded.

### Evaluation of cerebral infarct and edema volume

Mice were killed 24 h after ischemia, and brains were removed, snap frozen with liquid nitrogen and stored at −80°C. Thirty µm sections each separated by 420 µm spanning the infarct were cut using a cryostat (−21°C, CM1850, Leica Microsystems). Edema and corrected total infarct volumes were quantified as described [20]. Longitudinal distribution of the infarct was also calculated.

### Quantitative real time PCR

Brains were collected from sham-operated mice or at 6 or 24 h after ischemia, and the two hemispheres were separated and snap frozen in liquid nitrogen. RNA was extracted from the right (ischemic) hemisphere using Trizol reagent (Invitrogen, USA) and quantified. To assess the relative expression levels of mouse *Dscr1-1* (NCBI Reference Sequence; RefSeq NM_001081549.1) and *Dscr1-4* (RefSeq NM_019466.3), qRT-PCR was performed on cDNA samples using TaqMan Gene Expression Assays (Applied Biosystems, USA) and a real-time PCR system (ABI7900HT, Applied Biosystems). A Taqman Mouse Immune Array (Applied Biosystems) was used to assess the relative expression of selected immune-related genes.

### Immunohistochemistry

Mice subjected to ischemia were deeply anesthetized and transcardially perfused with PBS followed by 4% paraformaldehyde (PFA). Brains were removed and postfixed in 4% PFA for 24 h, then transferred into 30% (w/v) sucrose solution for a further 72 h, embedded in OCT (Tissue Tek, USA) and stored at −80°C. Twelve µm evenly spaced coronal sections separated by 380 µm were cut using a cryostat. The entire brain was sectioned in a series of 10 and mounted on Superfrost plus slides (Micromon, Walldorf, Germany). For each antibody, seven sections from one of the series encompassing the infarct region were immunoreacted with anti-rabbit Iba1 (1∶400; Wako, Japan; to detect macrophages and microglia) or anti-rat Ly-B.2 (1∶100; AbD Serotec, USA; to detect neutrophils). Appropriate secondary antibodies (1∶2000) were used (i.e. goat anti-rabbit Alexa Fluor 594 and goat anti-rat Alexa Fluor 488; Molecular Probes, USA). Stained sections were viewed with a fluorescent microscope (AxioImager Z1, Zeiss, Germany) using a 20× objective (Plan-Apochromat 20×/0.8 M27, Zeiss). Images were captured and analyzed using Image Pro Plus software. The number of neutrophils and microglia/macrophages within the brain was determined from the number of α-Ly-B.2- and α-Iba1-stained cells per mm^2^, respectively. The α-Iba1-stained cells were then identified as resting, activated or phagocytic based on their physical characteristics as described [23] and the proportions of each determined. For each section, four randomly selected fields within the infarct were captured and the entire field examined. For vWF immunofluorescence, 20 µm sections were incubated in an anti-rabbit vWF polyclonal antibody (1∶500, Abcam; USA), followed by a Texas Red-conjugated goat anti-rabbit secondary antibody (1∶200; Zymed Labratories, USA).

### Cortical neuronal studies

Primary cortical neuronal cultures were obtained from both WT and DSCR1-TG mice as described [24]. Briefly, cortices were dissected from mouse embryos at E15-16, and incubated for 15 min in 2 mg/ml trypsin in Ca^2+/^Mg^2+^-free Hank's balanced salt solution (HBSS; Invitrogen) buffered with 10 mM HEPES. The cells were plated at a density of 3×l0^4^ cells/cm^2^ and maintained at 37°C in Neurobasal medium containing B-27 supplements (Invitrogen), 2 mM L-glutamine, 0.001% gentamycin sulfate and 1 mM HEPES (pH 7.2). Cytosine arabinoside (Sigma-Aldrich, USA) was added to the culture to inhibit glial cell proliferation at 3 µM. Experiments were performed on 7 to 9-day-old cultures. Approximately 98% of the cells were neurons. For glucose deprivation, neurons were incubated in glucose-free Locke's medium containing (in mM) 154 NaCl, 5.6 KCl, 2.3 CaCl_2_, 1 MgCl_2_, 3.6 NaHCO_3_, 5 HEPES, pH 7.2, supplemented with gentamycin (5 mg/L; Invitrogen, USA) for 6, 12 or 24 h. Cell viability was assessed using trypan blue [25].

### Immunoblotting

Proteins were transferred onto a polyvinylidene fluoride membrane (Immobilon-P, Millipore, USA) and probed with the primary antibodies against the following: RCAN1 (Sigma-Aldrich, USA); β-tubulin (Chemicon, USA); calcineurin (Abcam, Cambridge, USA); β-actin (Sigma-Aldrich); cleaved caspase-3; p-AKT; p-P38; p-SAP/JNK; and p-P65 (Cell Signaling Technology, USA). Quantification was performed using ImageQuant software (GE healthcare, USA). Band intensities were normalized to β-tubulin or β-actin.

### Statistical analysis

All data are presented as mean ± SEM. Statistical analyses were performed using GraphPad Prism version 5 (GraphPad Software, USA). Between-group comparisons were made using a Student's unpaired *t* test or ANOVA with a Bonferroni or Newman-Keuls *post-hoc* test, as appropriate. Neurological score was compared using a Mann-Whitney test. Statistical significance was accepted when P<0.05.

## Results

### Over-expression of DSCR1 improves stroke outcome

**Figure 1:**
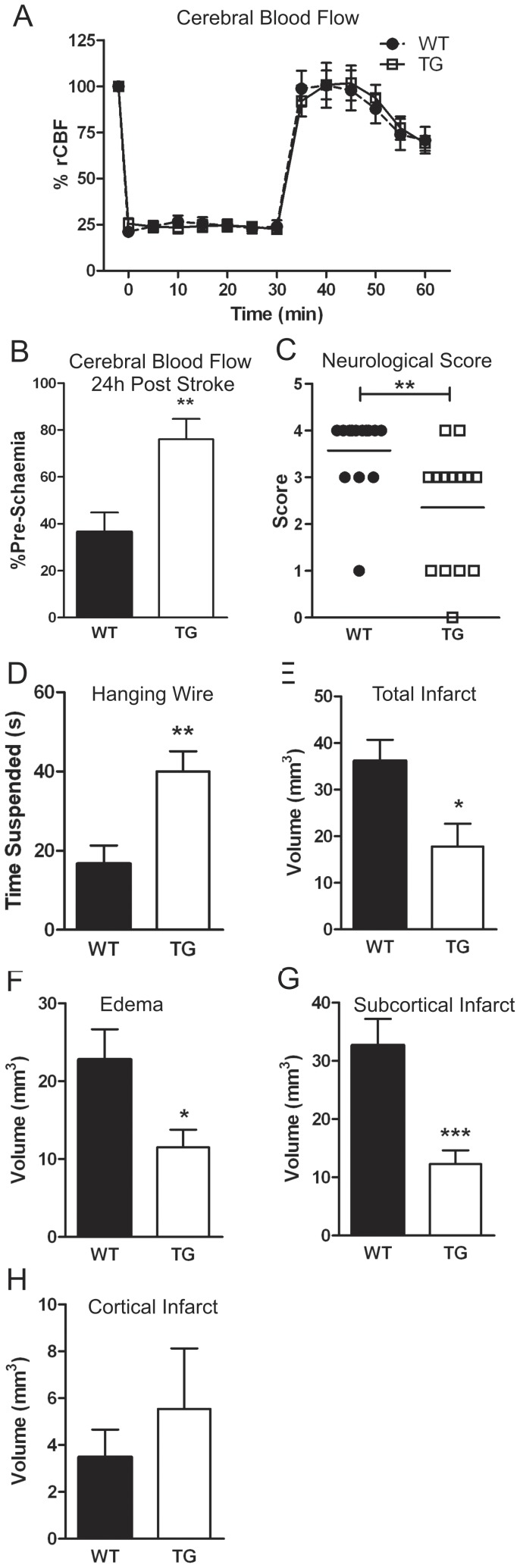
Over-expression of DSCR1 underlies an improved outcome following stroke. WT (black symbols and bars, n = 14) and DSCR1-TG (white symbols and bars, n = 14) mice were subjected to stroke. Regional cerebral blood flow (rCBF) was recorded during and for 0.5 h after stroke (A) and at 24 h (B), and expressed as a percentage of the pre-ischemic value. Compared with WT, DSCR1-TG mice had an improved neurological deficit score (C) and better performance in a hanging wire grip test (D). Total infarct (E) and edema (F) volumes were also smaller after stroke in DSCR1-TG than WT mice. DSCR1-TG mice had smaller subcortical (G) but not cortical (H) infarcts. Neurological scores are presented as median, and all other data are presented as mean ± SEM; *P<0.05, **P<0.01, ***P<0.001.

Systolic BP was similar in naïve DSCR1-TG and WT mice (TG, 129±3 mmHg; WT, 121±4 mmHg; *n* = 10–13). During ischemia, rCBF was reduced to ∼25% of the pre-ischemic level in each group, and was then increased similarly upon reperfusion (Fig. 1A). At 24 h, rCBF was 2-fold higher in DSCR1-TG than WT (Fig. 1B). DSCR1-TG mice had an improved neurological deficit score (*P*<0.05; Fig. 1C) and a greater grip strength (*P*<0.05; Fig. 1D) compared with WT. Both total infarct volume (edema-adjusted, *P*<0.05; Fig. 1E) and brain swelling (*P*<0.05; Fig. 1F) were ∼50% smaller in DSCR1-TG mice. The smaller infarct volume in DSCR1-TG was restricted to the subcortical regions of the brain (Fig. 1G–H; Fig. S1A). Infarct distribution was more rostral in DSCR1-TG mice than WT mice (Fig. S1B) and was not associated with any difference in brain vascularization between genotypes (Fig. S1C). Moreover, whereas hippocampal lesions were evident in 37/53 (70%) of sections examined from 11 WT mice, no hippocampal lesions were observed in >50 sections from 11 DSCR1-TG mice.

### Expression of DSCR1 isoforms and activity of calcineurin after ischemia

**Figure 2:**
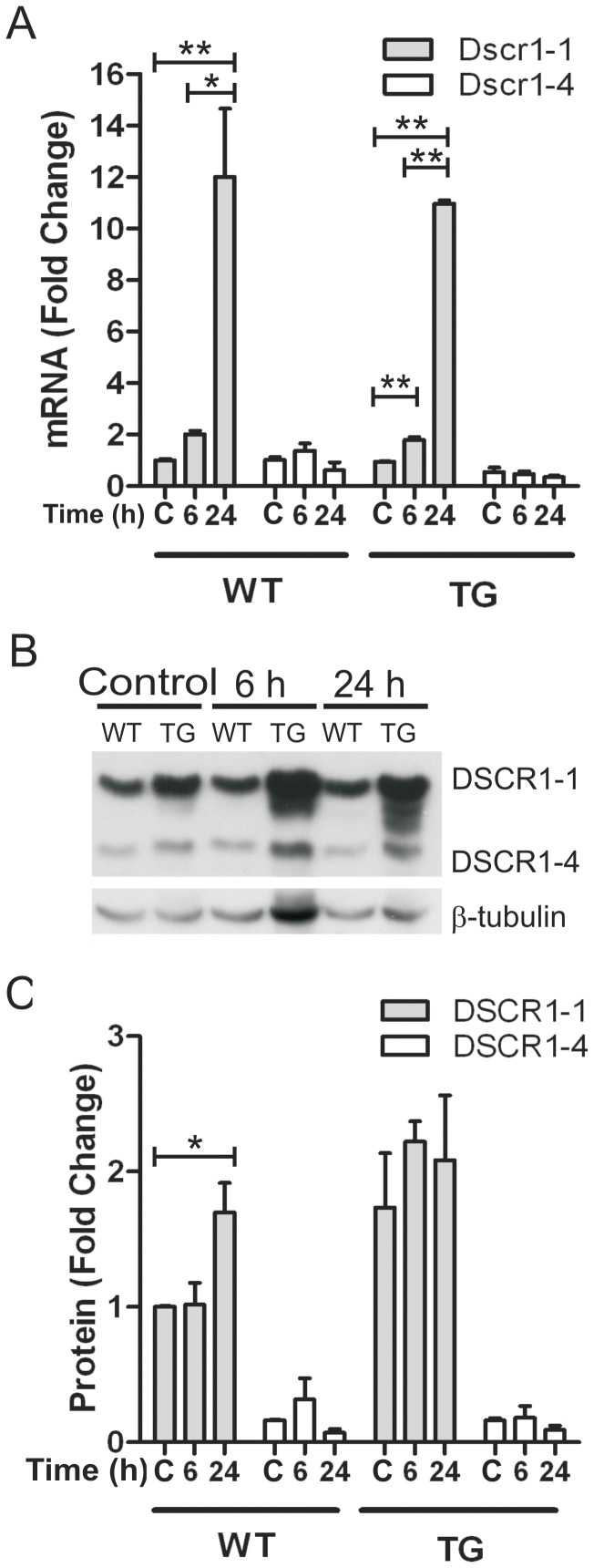
DSCR1 expression in the brain of WT and DSCR1-TG mice following stroke. qRT-PCR and Western blot analyses were used to assess the expression of DSCR1 isoform 1 (grey bars) and 4 (white bars) in WT and DSCR1-TG mice. Expression was assessed in naïve control mice [C] and in mice at 6 and 24 h after ischemia. (A) Endogenous *Dscr1-1* mRNA was increased at 6 and 24 h following stroke, although there was no difference between WT and DSCR1-TG. DSCR1-4 gene expression was not altered by ischemia in either group. (B and C) A representative Western blot is shown using an antibody that recognises both isoforms of DSCR1 protein, and cross-reacts with both mouse (endogenous) and human (transgene) proteins. DSCR1-1 protein levels were higher in DSCR1-TG compared with WT due to over-expression of the human DSCR1-1 transgene. DSCR1-1 protein was increased by ∼2-fold after ischemia in WT, but did not change after ischemia in DSCR1-TG. There was no effect of ischemia on DSCR1-4 protein expression in either genotype. The data are presented as mean ± SEM (n = 3 per group). (*P<0.05, **P<0.01).

**Figure 3:**
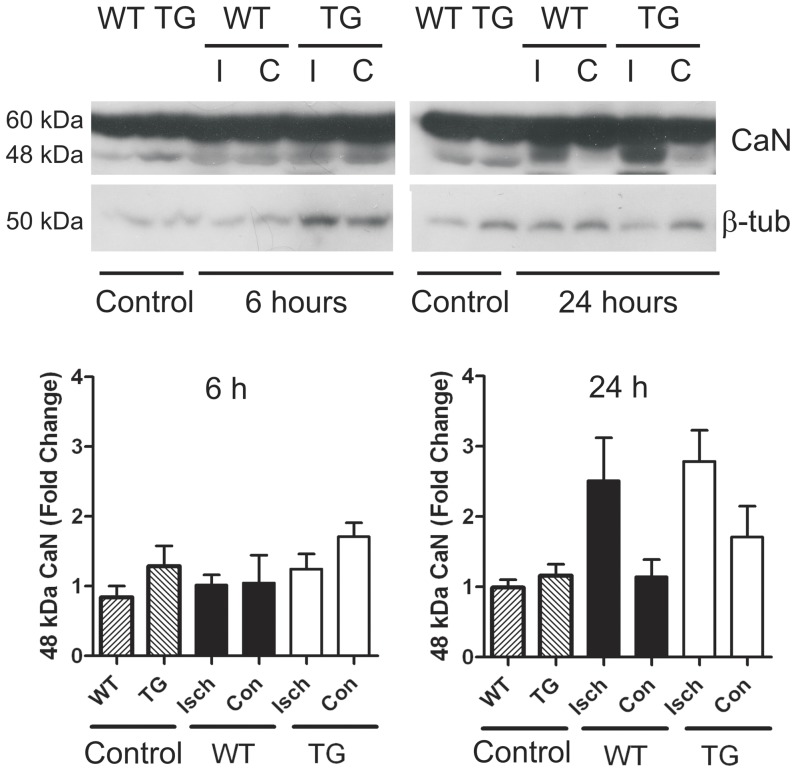
Over-expression of DSCR1 does not alter calcineurin (CaN) activity before or after stroke. Western blotting was used to determine the level of a constitutively active 48 kDa cleavage fragment of calcineurin that is produced upon calcineurin activation. Analysis of protein extracts from the ischemic (I, Isch) and contralateral (C, Con) brain hemispheres of naïve control (or post-stroke mice revealed that there was no difference in calcineurin activity between WT and DSCR1-TG either before or after stroke. Calcineurin activity was determined by normalizing the intensity of the 48 kDa band to β-tubulin. The data are presented as mean ± SEM (n = 3 per group).

Expression of endogenous *Dscr1-1* or *Dscr1-4* mRNA in naïve mice was not different between WT and DSCR1-TG (Fig. 2A), indicating that the human *DSCR1-1* transgene did not affect expression of either endogenous *Dscr1* isoform. However, mouse *Dscr1-1* mRNA was increased by ∼12-fold at 24 h in the ischemic hemisphere of both WT and DSCR1-TG (Fig. 2A). By contrast, expression of *Dscr1-4* mRNA was not altered by ischemia (Fig. 2A). Over-expression of the human *DSCR1-1* transgene resulted in ∼2-fold higher levels of total DSCR1-1 protein in the brain of naïve DSCR1-TG compared with WT mice, whereas prior to ischemia there were similar levels of DSCR1-4 protein in WT and DSCR1-TG (Fig. 2B–C). Cerebral ischemia had no significant effect on either DSCR1-1 or DSCR1-4 protein expression in DSCR1-TG mice, whereas DSCR1-1 protein was elevated in WT at 24 h (Fig. 2C). In brains from both WT and DSCR1-TG mice, calcineurin activity was unchanged at 6 h, but it was increased by 2–3 fold in the ischemic hemisphere at 24 h (Fig. 3). Notably, at no time point was any difference detected in calcineurin activity between WT and DSCR1-TG (Fig. 3).

### Inflammation following cerebral ischemia

**Figure 4:**
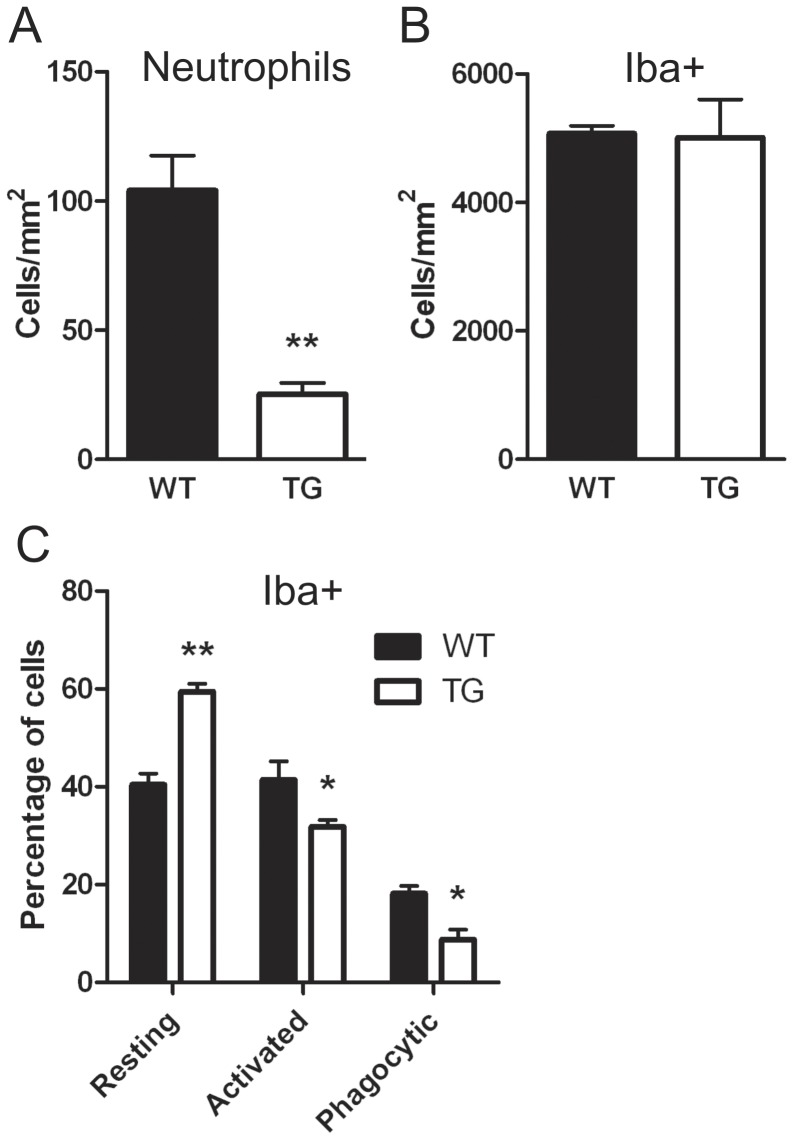
DSCR1-TG mice exhibit reduced brain inflammation following stroke. The number of neutrophils was significantly reduced in DSCR1-TG versus WT brains following stroke (A). While there was no difference in the density of Iba+ cells between DSCR1-TG and WT mice (B), more of these cells were in a resting state and fewer in activated or phagocytic states in DSCR1-TG (C). The data are presented as mean ± SEM (n = 3 per group). (*P<0.05, **P<0.01).

**Figure 5:**
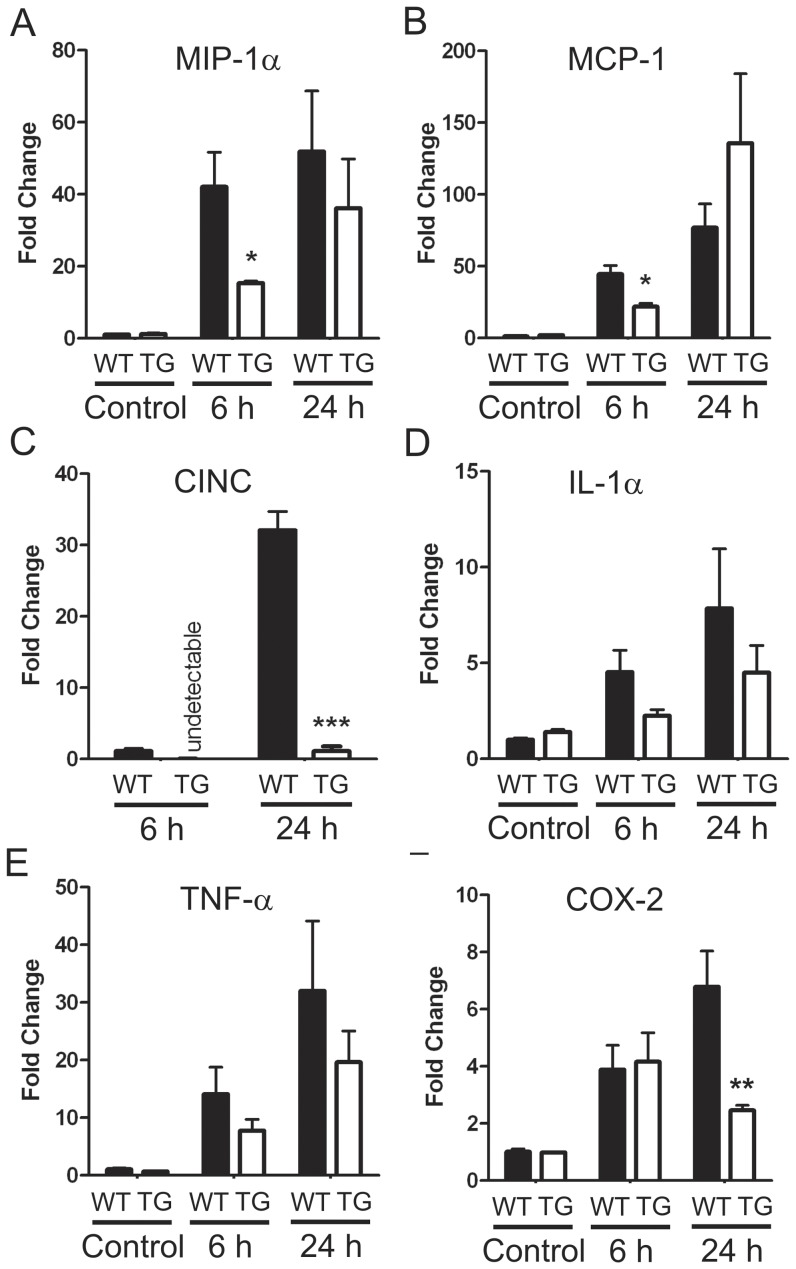
DSCR1-TG mice exhibit a reduction in inflammatory mediators following stroke. Cerebral ischemia results in increased mRNA levels for several inflammatory mediators in the brain over 24 h, including (A) *MIP-1α*, (B) *MCP-1*, (C) *CINC*, (D) *IL-1α*, (E) *TNF-α* and (F) *COX-2*. Of note, expression of *MIP-1α* (A) and *MCP-1* (B) was delayed in DSCR1-TG, while *CINC* expression was virtually unchanged (C). *COX-2* expression was reduced in the DSCR1-TG at 24 h after ischemia (F). The data are presented as mean ± SEM (n = 3 per group). (*P<0.05, **P<0.01, ***P<0.001).

The influx of leukocytes is a major effector of inflammatory damage following experimental stroke ([26]. Over-expression of DSCR1 was associated with 80% fewer neutrophils infiltrating the ischemic hemisphere at 24 h post-stroke compared with WT mice (Fig. 4A). On average, RCAN1-TG mice had 25.25±4.47 neutrophils per unit area compared to 104.1±13.48 in the WT. Furthermore, while the total number of microglia/macrophages (i.e. Iba-1^+^ cells) in the ischemic hemisphere was similar in the two groups at 24 h (Fig. 4B), more of these cells were in the resting state in DSCR1-TG brains (58.40±1.64% versus 40.42±2.25% in WT), and fewer were in activated (31.83±1.34% in TG versus 42.41±3.75% in WT) or phagocytic (8.76±2.02 in TG versus 18.16±1.51% in WT) states (Fig. 4C). T lymphocytes were not detected in brains of either group at 24 h. Consistent with these data, over-expression of DSCR1 inhibited the upregulation of several genes related to inflammation following stroke (Fig. 5). At 6 h, expression of mRNA for macrophage inflammatory protein-1α (*MIP-1α/Ccl3*), monocyte chemoattractant protein-1 (*MCP-1/Ccl2*), and cytokine-induced neutrophil chemoattractant (*CINC*) were increased in WT and were significantly higher than in DSCR1-TG (Fig. 5A–C). *CINC* expression was undetectable in naïve animals of both genotypes and thus results are expressed relative to WT at 6 h (Fig. 5C). Levels of *IL-1α* and *TNF-α* tended to be higher in WT than DSCR1-TG at both 6 h and 24 h (Fig. 5D–E), whereas the level of *COX-2* mRNA was significantly higher in WT than DSCR1-TG at 24 h (Fig. 5F).

### Neuronal apoptosis following glucose deprivation

**Figure 6:**
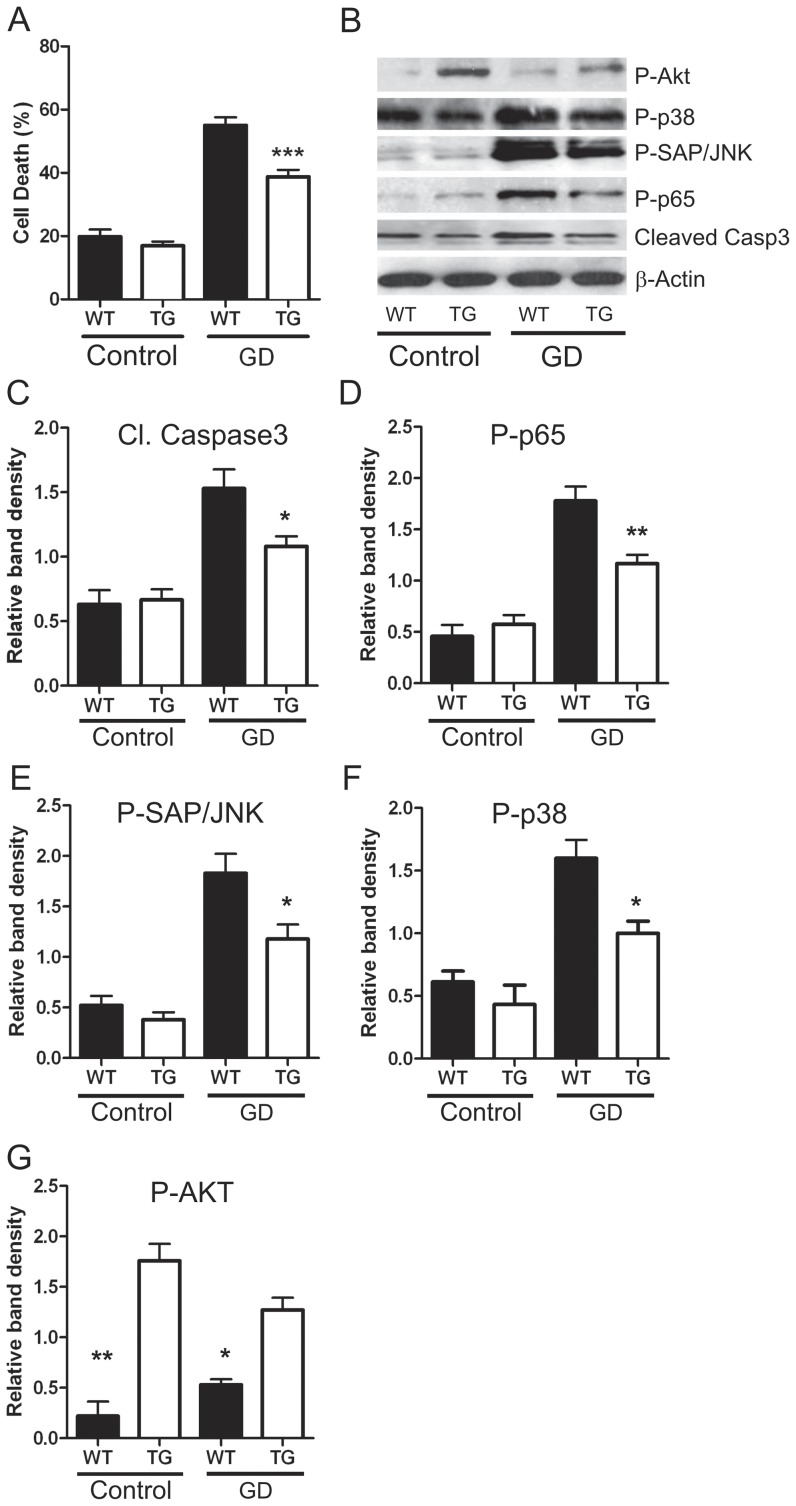
Over-expression of DSCR1 is protective in neurons during glucose deprivation (GD) *in vitro*. DSCR1-TG neurons (white bars) exhibited an improved survival compared with WT (black bars) following 24 h exposure to GD (A). This protection was preceded at 12 h by reduced expression of cleaved caspase-3, P-p65, P-SAP/JNK and P-p38 MAPK (B–F). P-AKT was higher in DSCR1-TG than WT neurons under control conditions and this difference was maintained after GD (B,G). Data are presented as mean ± SEM; *P<0.05, **P<0.01, ***P<0.001.

To examine the effects of excess DSCR1 in neurons subjected to a cell culture model of ischemia, dissociated cortical neurons were subjected to glucose deprivation (GD) [25]. Neurons isolated from DSCR1-TG mice were more resistant than WT neurons to apoptotic cell death following 24 h of glucose deprivation *in vitro* (Fig. 6A). This was preceded at 12 h by reduced expression of cleaved caspase-3, phospho-p65, phospho-SAP/JNK and phospho-p38 MAPK (Figs. 6B–F). By contrast, expression of the pro-survival protein, phospho-AKT, was higher in DSCR1-TG versus WT neurons under control conditions and this difference was maintained after 12 h of glucose deprivation (Fig. 6G).

## Discussion

This is the first study to investigate the effect of *DSCR1* gene over-expression on outcome following ischemic stroke. We report that the over-expression of human *DSCR1* in mice reduces neurological deficit, cerebral infarct volume and brain swelling following stroke. The results suggest that this protective effect may occur through two main mechanisms. Firstly, attenuated upregulation of several pro-inflammatory genes and as less neutrophil infiltration and microglial activation in DSCR1-TG mice suggest that *DSCR1* over-expression exerts an anti-inflammatory effect to limit brain injury following stroke. Secondly, neuronal culture experiments revealed that DSCR1-TG neurons display a greater resistance to apoptotic cell death following glucose deprivation, consistent with neuronal expression of DSCR1 contributing to improved post-stroke outcome.

Over-expression of *DSCR1* in our DSCR1-TG mice occurs under the direction of its own promoter [19]. Strong DSCR1 immunoreactivity is normally present in the striatum and the CA1 and CA3 pyramidal cells of the hippocampus [9], i.e. areas directly affected by ischemia in our model. Thus, it is plausible that neuronal cells of the transgenic mice were directly protected from ischemic injury. It was particularly noteworthy that in spite of the well known sensitivity of the hippocampus to damage by hypoxia [27], we observed no hippocampal lesions in DSCR1-TG mice.

We evaluated the effect of *DSCR1* over-expression in a cell culture model of neuronal injury in which primary mouse cortical neurons were subjected to ischemia-like conditions of glucose deprivation. Indeed, cell death was markedly reduced in neurons from DSCR1-TG versus WT mice, and this was associated with an attenuated increase in the level of cleaved caspase-3, an apoptotic protease. Moreover, levels of activated NFκB, SAP/JNK and p38-MAPK were markedly increased by glucose deprivation in WT neurons, whereas their levels were significantly lower in DSCR1-TG. Activation of these signaling pathways has been negatively associated with ischemia-induced cellular stress responses and neuronal death [28]. Our data therefore suggest that DSCR1 over-expression in neurons protects against oxidative injury and apoptotic cell death by limiting activation of these key pathways. Notably, the pro-survival factor, p-AKT, was present at elevated levels in DSCR1-TG neurons.

DSCR1-1 mRNA and protein are more abundant than DSCR1-4 in the brains of humans and mice [8], [9]. We found evidence for a small increase in *Dscr1-1* mRNA expression at 6 h and a much greater increase at 24 h after ischemia in both genotypes. Total (i.e. human and mouse) DSCR1-1 protein was ∼2-fold higher in DSCR1-TG than in WT. Nevertheless, DSCR1-1 protein expression only increased modestly in the WT after ischemia, and was not changed after ischemia in DSCR1-TG. Thus, the neuroprotection due to *DSCR1-1* over-expression was not related to a further change in DSCR1 protein levels following stroke. DSCR1-4 expression has been reported to increase in the brains of rats and mice after ischemia [13], [14]. In contrast, in the present study we found no change in DSCR1-4 mRNA or protein expression after ischemia, although the reason for this discrepancy remains unknown.

Our findings demonstrate that smaller infarcts develop in DSCR1-TG mice compared with WT despite both genotypes receiving an equivalent ischemic insult. A higher rCBF detected in DSCR1-TG at 24 h may in part reflect improved brain perfusion due to less swelling and consequently lower intracranial pressure and/or a reduction in the ‘no-reflow phenomenon’ due to obstruction of capillaries by leukocytes. After stroke, leukocytes are recruited to the site of injury by chemokines and release cytokines and other pro-inflammatory molecules which potentiate brain damage [29]. Microglia, the resident population of macrophages located within the brain, are also activated and this also leads to the release of inflammatory mediators [29]. Over-expression of *DSCR1* resulted in less microglial activation and less influx of neutrophils following ischemia, in association with lower levels of inflammatory mediators. This reduction in microglial activation and leukocyte infiltration may protect the infarcted DSCR1-TG brain from secondary damage caused by inflammation. Indeed, inhibiting the influx of monocytes/macrophages into the brain results in a smaller infarct size and improved outcome following ischemia [30], [31], and inhibition of neutrophil infiltration through administration of an anti-neutrophil antibody prior to the induction of stroke, may also result in a reduced infarct volume [32]–[34]. Of particular note, expression of *CINC* was markedly lower in DSCR1-TG mice after ischemia, which is likely to have contributed to the reduced number of infiltrating neutrophils.

Calcineurin, which stimulates NFAT-mediated transcription of pro-inflammatory genes [16], is highly expressed in regions of brain that are most susceptible to ischemia, and high levels of calcineurin activity predispose neurons to apoptosis [35]. Administration of pharmacological inhibitors of calcineurin, which are powerful immunosuppressants used to prevent rejection of transplanted organs, are reported to reduce lesion size and neurological deficit following stroke [18]. However, although we found calcineurin activity to be increased in the ischemic hemispheres of both DSCR1-TG and WT mice at 24 h, the level of activity was not different between the genotypes suggesting that the protective effects of DSCR1 are unlikely to be mediated by suppression of calcineurin activity.

Finally, upregulation of DSCR1 has been suggested to contribute to parts of the Down syndrome phenotype [36], [37]. Indeed, it seems that elevated levels of DSCR1 are responsible, at least in part, for a decrease in the incidence of solid tumors in Down syndrome [36], suggesting that over-expression of DSCR1 is protective. Interestingly, with the exception of Moyamoya syndrome, caused by stenosis of the bilateral supraclinoid internal carotid arteries, which has been occasionally but infrequently associated with Down syndrome [38], there is a dearth of reports on stroke in the Down syndrome population. This is particularly striking when one considers that Down syndrome is often described as a disorder of precocious aging, and that advancing age is associated with an increased incidence of stroke. It is therefore possible that excess DSCR1 provides some degree of protection against stroke.

In summary, our study provides the first evidence that DSCR1 over-expression improves functional and histological outcomes following stroke. Our data are consistent with the mechanisms underlying this protection involving anti-inflammatory and anti-apoptotic effects mediated by DSCR1.

## Supporting Information

Figure S1 **Representative coronal brain sections are shown from WT and DSCR1-TG mice 24 h after stroke at bregma -0.08 mm (A, upper panels), -1.76 mm (A, middle panels) and -3.44 mm (A, lower panels), with the infarct area outlined in white.** Profile of brain infarct location in WT (black symbols) and DSCR1-TG (white symbols) following stroke (B). Representative photomicrographs taken from bregma -1.76 mm, showing similar von Willebrand Factor (vWF) immunofluorescent staining within the right hemisphere of a naïve WT (C, left panel) and DSCR1-TG (C, right panel) mouse. The scale bar represents 100 µm.
(TIF)
