# Supplementary figures and images for: Over-Expression of DSCR1 Protects against Post-Ischemic Neuronal Injury
Source: PLoS One. 2012 Oct 29;7(10):e47841. doi: 10.1371/journal.pone.0047841 (PMC3483156; doi:10.1371/journal.pone.0047841)

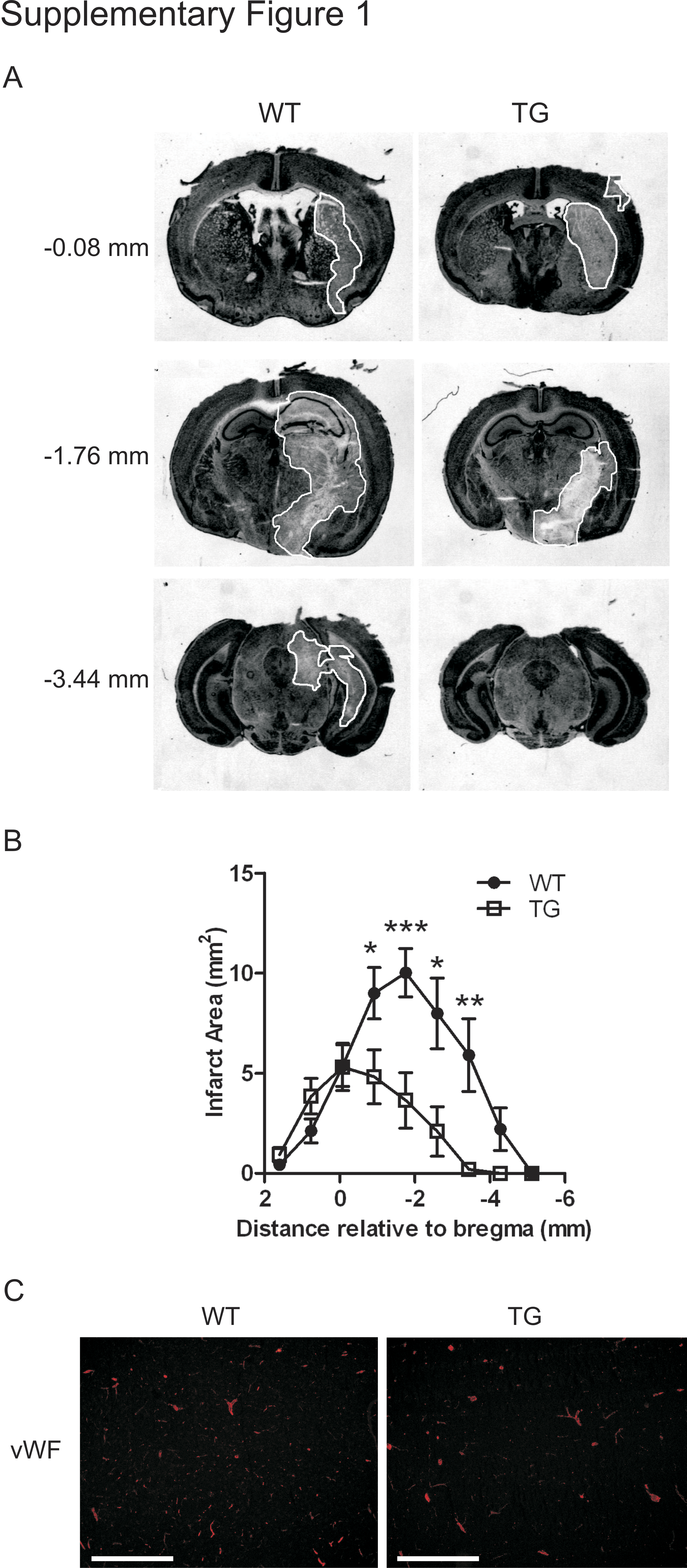

Supplement: Figure S1 — Representative coronal brain sections are shown from WT and DSCR1-TG mice 24 h after stroke at bregma -0.08 mm (A, upper panels), -1.76 mm (A, middle panels) and -3.44 mm (A, lower panels), with the infarct area outlined in white. Profile of brain infarct location in WT (black symbols) and DSCR1-TG (white symbols) following stroke (B). Representative photomicrographs taken from bregma -1.76 mm, showing similar von Willebrand Factor (vWF) immunofluorescent staining within the right hemisphere of a naïve WT (C, left panel) and DSCR1-TG (C, right panel) mouse. The scale bar represents 100 µm. (TIF) [file pone.0047841.s001.tif]
